# The role of hand hygiene in controlling norovirus spread in nursing homes

**DOI:** 10.1186/s12879-016-1702-0

**Published:** 2016-08-09

**Authors:** Rania Assab, Laura Temime

**Affiliations:** Laboratoire MESuRS, Conservatoire National des Arts et Métiers, 292 Rue Saint-Martin, 75003 Paris, France

**Keywords:** Nursing homes, Mathematical modeling, Infection control, Norovirus, Hand hygiene

## Abstract

**Background:**

Norovirus, the leading cause of gastroenteritis, causes higher morbidity and mortality in nursing homes (NHs) than in the community. Hence, implementing infection control measures is crucial. However, the evidence on the effectiveness of these measures in NH settings is lacking. Using an innovative data-driven modeling approach, we assess various interventions to control norovirus spread in NHs.

**Methods:**

We collected data on resident and staff characteristics and inter-human contacts in a French NH. Based on this data, we developed a stochastic compartmental model of norovirus transmission among the residents and staff of a 100-bed NH. Using this model, we investigated how the size of a 100-day norovirus outbreak changed following three interventions: increasing hand hygiene (HH) among the staff or residents and isolating symptomatic residents.

**Results:**

Assuming a baseline staff HH compliance rate of 15 %, the model predicted on average 19 gastroenteritis cases over 100 days among the residents, which is consistent with published incidence data in NHs. Isolating symptomatic residents was highly effective, leading to an 88 % reduction in the predicted number of cases. The number of expected cases could also be reduced significantly by increasing HH compliance among the staff; for instance, by 75 % when assuming a 60 % HH compliance rate. While there was a linear reduction in the predicted number of cases when HH practices among residents increased, the achieved impact was less important.

**Conclusions:**

This study shows that simple interventions can help control the spread of norovirus in NHs. Modeling, which has seldom been used in these settings, may be a useful tool for decision makers to design optimal and cost-effective control strategies.

## Background

Combined with community life and limited resources, the increased vulnerability of older adults leads to a high prevalence of infections in nursing homes (NHs), with major consequences in terms of morbidity, mortality and costs [1]. In particular, norovirus gastroenteritis is one of the most frequent causes of outbreaks in NH settings [2], leading to increased death and hospitalization rates; for instance, a US study showed that long-term care residents were four times more likely to die from gastroenteritis than people living in the community [3].

Controlling norovirus outbreaks has proved extremely difficult in the past, due to the high transmissibility of the virus, its environmental persistence and its prolonged shedding in previously infected individuals [4, 5]. In NHs, this is added to by the lack of infection control recommendations specifically adapted to these settings. In particular, as person-to-person transmission plays an important part in norovirus spread and compliance with international hygiene recommendations is low in NH staff [6], it is expected that increasing hand hygiene practices could help limit norovirus outbreaks. However, the evidence on hand hygiene effectiveness in NH settings is limited [7] and the way hand hygiene may impact norovirus transmission dynamics is poorly understood.

Mathematical models are useful tools to help understand the propagation of an epidemic and assess control strategies; however, to this date, very few models of norovirus spread have been proposed, none of which was specific to NH settings [8, 9]. In this context, our objective was to investigate the impact of increasing hand hygiene compliance in NH staff and/or residents on the risk of norovirus gastroenteritis outbreaks in a NH, using a mathematical modeling approach.

## Methods

### Model of norovirus transmission

**Fig. 1:**
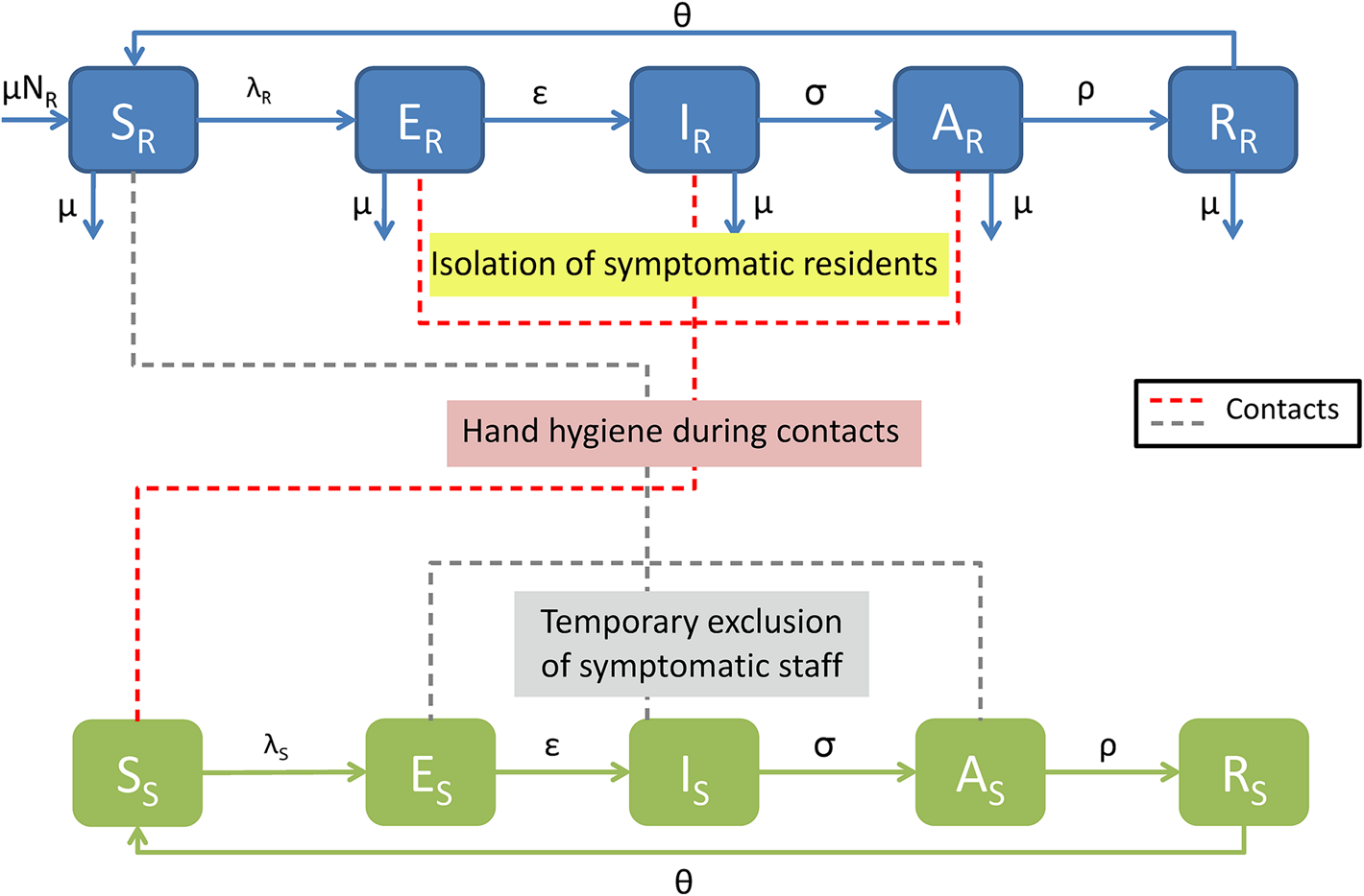
Stochastic model of norovirus transmission in a nursing home. Residents (resp. the staff) are designated by the subscript R (resp. S). Individuals may be classified as susceptible to norovirus infection (S), exposed to norovirus but not yet symptomatic (E), infected and symptomatic (I), infected but asymptomatic (A), or immune (R)

We simulated norovirus spread in a 100-bed NH. To model norovirus transmission in the NH, we developed a stochastic compartmental SEIAR model [8], in which the human population was divided into several compartments according to their infection status: susceptible individuals (S), exposed but not yet symptomatic individuals (E), infected and symptomatic individuals (I), infected but asymptomatic individuals (A), and immune individuals (R) (Fig. 1). Two different sub-populations were taken into account: the residents and the NH staff.

Residents were admitted to the NH at a rate μ, which was assumed to be equal to the rate of resident discharge or death. Only direct person-to-person norovirus transmission was included in the model; foodborne contamination was not taken into account. Susceptible (S) individuals could acquire norovirus via a contact with an infected (I) individual with a given probability; this per-contact transmission probability scaled with contact duration and depended on the nature of the contact (staff-staff, resident-resident, etc.). The probability p of norovirus transmission over a 15-min contact between a nurse and a resident was used as reference. Pre-symptomatic and asymptomatic individuals have been observed to transmit norovirus [10]; in a recent modeling study, their infectiousness was estimated at 5 % that of symptomatic individuals [8]. Hence, we assumed that norovirus could also be transmitted by pre-symptomatic (E) and asymptomatic (A) individuals, but with a probability reduced by a factor α. After norovirus acquisition, individuals went through a latent phase for a duration 1/ε before developing symptoms; symptoms lasted for a duration 1/σ on average, but norovirus shedding could persist for an additional 1/ρ duration. After full recovery, immunity was lost at a rate θ. Infected staff had a probability η of presenting enough symptoms to be detected, in which case they were assumed to be sent home until the end of their symptoms.

Model parameters are listed in Table 1, along with their assumed values and the list of the stochastic transitions along with their rates are shown in Appendix.

**Table 1:** Model parameters: baseline values and investigated ranges

Parameters			Assumed value		
			Baseline	Range	Reference
No. of staff			63	35–85	Collected data
No. of residents			100	59–118	Collected data
Admission and discharge rate		μ	2 %/month	0.01–0.25	Collected data
Transmission probability		p	0.08/contact	0.03–0.18	Calibrated baseline + [15]
Detection rate of infected staff		η	0.68	0.32–1	[16]
Contact rate	Staff-Staff	C_SS_	0.045/day	0–0.1	Collected data
	Staff-Resident	C_SR_	0.1/day	0.08–0.17	Collected data
	Resident-Resident	C_RR_	0.025/day	0–0.15	Collected data
Relative infectiousness of A and E		α	0.05	0–0.1	[8]
Duration of incubation		1/ε	1 day	-	[15]
Duration of symptoms		1/σ	2 days	1–3	[17]
Duration of asymptomatic phase		1/ρ	10 days	-	[18]
Duration of immunity		1/θ	5.01 years	4.0–6.7	[8]
Hand hygiene compliance rate during interactions	Staff-Staff	h_SS_	0	0–0.5	Collected data
	Staff-Resident	h_SR_	0.15	0.05–0.6	[6, 19, 20]
	Resident-Resident	h_RR_	0.1	0–0.5	Collected data

### Contact data

In May 2015, we conducted a questionnaire survey in the “Jardins d’Alésia” nursing home, located in Paris (France). This NH offers 102 beds, all in single rooms, and hosted 100 residents at the time of our survey. Using the questionnaire, which was administered by RA to both the nursing home director and the nurse manager, we collected detailed data on inter-individual contacts typically occurring within the NH, including:

- Number and duration of the daily contacts of a given resident with each staff category
- Number and duration of the daily contacts of a given staff member with other staff members
- Number and duration of the daily contacts of a given resident with other residents
- Nature and risk level of all contacts

This data was used to compute contact rates for the model. Table 2 summarizes the findings from this survey and our contact rates computations.

**Table 2:** Contact rates within the nursing home: collected data and rates used in the model

A- Contacts between staff members and residents				
Staff category	Mean no. of contacts/day/resident	Mean duration of contacts (min)	Risk level of contacts	Weighted no. of contacts/day
Nurse	1	6.5	1	0.44
Auxiliary nurse	2	15 ^a^	1^a^	2
Receptionist	1	1	0.5	0.04
Activity leader	1	20	0.5	0.67
Ancillary staff (e.g. catering staff)	2	40	0.5	2.67
Therapist	1	10	0.5	0.34
Building maintenance staff	1	5	0.5	0.17
Total weighted number of contacts/resident/day				6.33
Computed Staff-Resident daily contact rate C_SR_				6.33/63 = 0.1
B- Contacts between staff members				
Average proportion of the working staff contacted over a given day, per staff member		Proportion of staff working on any given day in the NH	Risk level of contacts	Computed Staff-Staff daily contact rate C_SS_
90 %		21 %	0.25	0.045
C- Contacts between residents				
Mean no. of contacts/day/resident	Risk level		Weighted no. of contacts/day	Computed Resident-Resident daily contact rate C_RR_
5	0.5		2.5	2.5/100 = 0.025

^a^Taken as reference

For a given resident, the average number of daily contacts with staff members is provided for each staff category (nurses, auxiliary nurses, cleaners…), along with the average duration of these contacts and their assumed relative risk level for norovirus transmission as compared to that of a nurse-resident contact (Table 2A). Based on this data, we computed C_SR_, the staff-resident daily contact rate used in the model, as follows. First, we computed a weighing factor R_c_ for each contact as: R_c_ = $\frac{{\mathrm{D}}_{\mathrm{c}}}{15}$ ×RL_c_ where D_c_ is the contact duration (in minutes) and RL_c_ is the assumed risk level of the contact. Secondly, we determined the at-risk contact rate C_SR_ between residents and staff as the weighted average of all daily numbers of staff-resident contacts:

$$ {\mathrm{C}}_{\mathrm{SR}}={\displaystyle {\sum}_{resident- staff\; contacts}}\frac{N_c\times {R}_c}{N_{staff}} $$

Here, for a given staff-resident contact type, N_c_ is the average daily number of occurrences of this contact per resident, R_c_ is the weighing factor computed earlier and N_staff_ is the total number of staff employed by the NH (N_staff_ = 63). For instance, based on the collected data, each day a resident had two contacts on average with members of the catering staff, each of which lasted approximately 40 min, with an assumed risk level for norovirus transmission 50 % that of a nurse-resident contact. Hence, this particular staff-resident contact type was imputed into the global C_SR_ computation as: 2 × (40/15) × 50 % = 2.67 at-risk contacts per day and per resident. In total, there were 6.33 at-risk contacts with the NH staff per day and per resident, leading to an at-risk staff-resident contact rate of: C_SR_ = 6.33/63 = 0.10 / day.

The survey showed a highly variable duration of staff-staff and resident-resident contacts. Hence, we based our computation of the corresponding contact rates on three factors: the observed numbers of individuals in contact per day, the assumed risk level of these contacts and the total headcounts of residents or staff working in the NH (Table 2B and C). For staff-staff contacts, the probability of being at work on a given day was also taken into account; based on 35-h work weeks, it was computed as 21 % (Table 2B). The resulting at-risk contact rates were C_SS_ = 0.045/day and C_RR_ = 0.025/day.

### Model fitting and simulations

#### Numerical simulations

We performed simulations over 100 days, assuming the admission at day 1 of two infected residents in the NH. The simulations were performed using Gillespie’s direct method [11]. For each modeled scenario, we ran 4,000 simulation replicates over which we computed the average of model outcomes.

The model was implemented and simulation results were analyzed using R version 3.2.3 [12], a free software environment for statistical computing. We used basic packages such as “stats” and “graphics”. For the figures layout, the package “ggplot2” was used.

#### Model fitting

The model was fitted to data from a published systematic review of gastro-enteritis prospective surveillance in long-term care facilities [13]. Using a least-square criterion, the per-contact transmission probability p and the relative infectiousness α of A and E individuals were calibrated so that the average 100-day cumulated incidence, computed over 4,000 simulations of the model, best reproduced the mean cumulated incidence that was reported in this meta-analysis.

#### Explored scenarios

We evaluated the impact of hand hygiene on norovirus spread by computing the predicted cumulated number of cases among residents over 100 days as a function of three distinct hand hygiene compliance measures:

1. Compliance to hand hygiene of the NH staff during their contacts with residents
2. Compliance to hand hygiene of the NH staff during their contacts with each other
3. Compliance to hand hygiene of NH residents during their contacts with each other

In all three cases, hand hygiene was modeled by reducing the transmission probability during contact type C by a factor (1-h_C_), where h_C_ was the hand hygiene compliance involved in that contact. At baseline, compliance rates were rather low, as suggested by observational studies in NHs, but values up to 50 or 60 % were investigated for this exploratory study (Table 1). Here, hand hygiene was defined as hand rubbing with an alcohol-based solution right before the contact took place.

In a second step, we simulated the impact of infected resident isolation by modifying the contact rate of infected residents. We assumed that isolated residents had no contacts with other residents (C_RR_ = 0) and that their contacts with the staff were limited to those with healthcare workers (nurses, auxiliary nurses and physicians), leading to a reduced value of C_RP_ (0.04/day). We then computed the predicted cumulated number of norovirus infection cases among residents over 100 days under this scenario, and compared the effectiveness of a control strategy based on increasing hand hygiene compliance to the effectiveness of case isolation.

#### Sensitivity analyses

In order to evaluate the impact of each parameter on model predictions (predicted total number of cases over 100 days), univariate and multivariate sensitivity analyses were undertaken. The parameters were allowed to vary within a range of their possible values (Table 1). Latin Hypercube Sampling (LHS), generated with the “lhs” package, was used and partial rank correlation coefficients (PRCC) were computed wth the “epiR” package for all parameters [14].

## Results

### Model calibration and baseline predictions

The reported incidence in the review by Kirk [13] was 0.40 gastroenteritis episodes per 1000 bed-days (95 % confidence interval: 0.27–0.56). Assuming that a single gastroenteritis epidemic occurs each year, as is the case in most developed countries, we translated this incidence as an average 14.6 cases (9.9-20.4) per outbreak in a 100-bed NH. The reference per-contact transmission probability and relative infectiousness of Exposed and Asymptomatic individuals were then calibrated at *p* = 0.08 / contact and α = 5 %. Using these calibrated values, under the baseline scenario, admission of two infected residents at day 1 led to a total of 19 cases over 100 days on average. After 100 days, the incidence was very low.

**Fig. 2:**
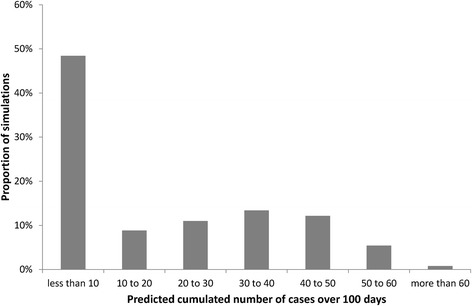
Distribution of the predicted number of cases over 100 days among 4,000 stochastic simulations

**Fig. 3:**
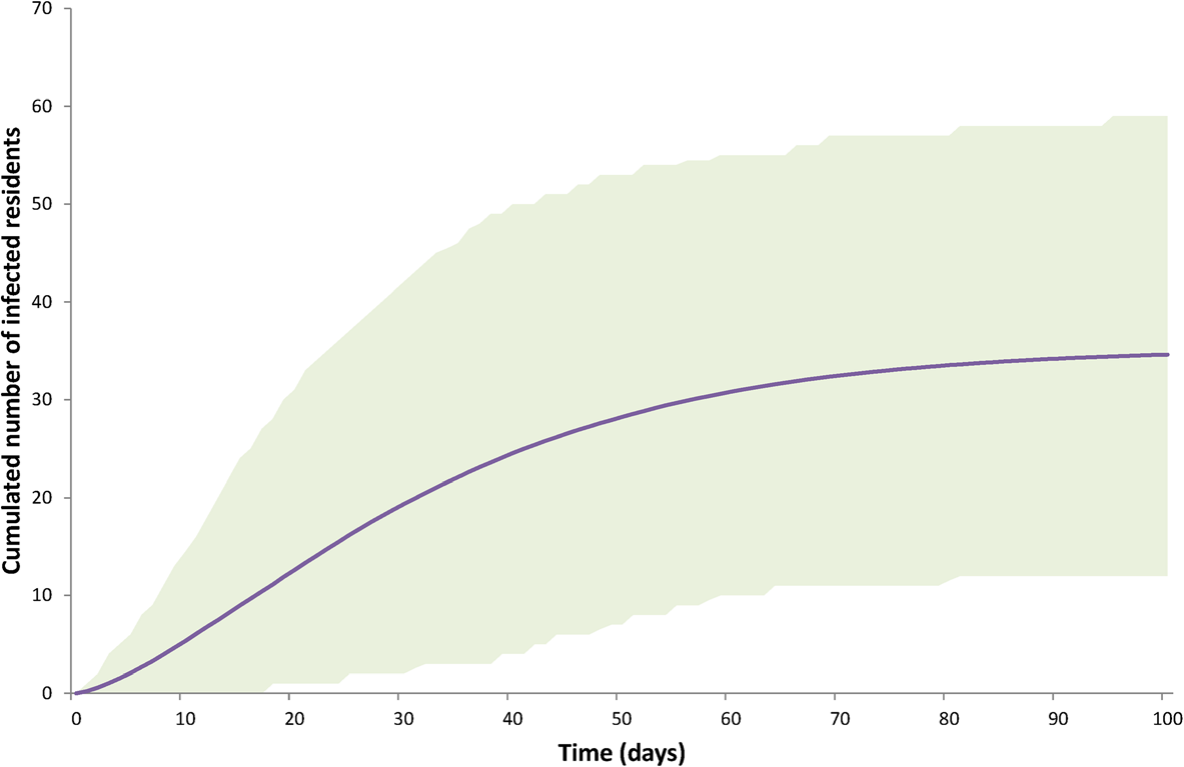
Cumulated predicted norovirus incidence among the residents over 100 days following the admission of two infected residents: mean (line) and 95 % prediction bands (shaded area) based on simulations in which at least 10 cases occurred

Due to stochastic extinctions, the distribution of epidemic sizes over 100 days was bimodal, with no secondary norovirus infection in 20 % of simulations, and 48 % of simulations leading to less than 10 cases of norovirus infection among residents (Fig. 2). Figure 3 provides the predicted cumulated incidence as a function of time, together with the prediction bands, for the simulations in which at least 10 secondary cases occurred. In the following sections, presented results are based on all scenarios, including those in which stochastic extinction occurs.

### Impact of hand hygiene compliance

**Fig. 4:**
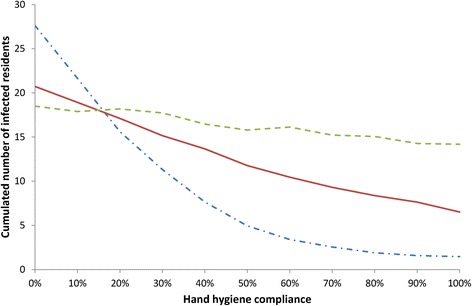
Impact of hand hygiene (HH) compliance on norovirus spread: predicted cumulated number of norovirus infection cases among residents over 100 days as a function of HH compliance of staff members during their contacts with residents (dash-dot line), HH compliance of staff members during contacts with other (dashed line) and HH compliance of residents during contacts with other (full line)

Figure 4 depicts the predicted average number of norovirus cases among residents over 100 days as a function of the three considered measures of hand hygiene compliance: compliance of NH staff during their contacts with residents (h_SR_), compliance of residents during their contacts with each other (h_RR_), and compliance of NH staff during their contacts with each other (h_SS_).

The compliance of hand hygiene of the staff during their contacts with residents had a major impact on norovirus propagation. The predicted average epidemic size decreased sharply when this compliance increased up to approximately 60 %; for higher compliance rates, the decrease was slower.

A linear decrease in predicted cases was also noted with increasing compliance among residents while hand hygiene of the staff during their contacts with each other did not impact norovirus propagation significantly.

### Comparison of control strategies

**Fig. 5:**
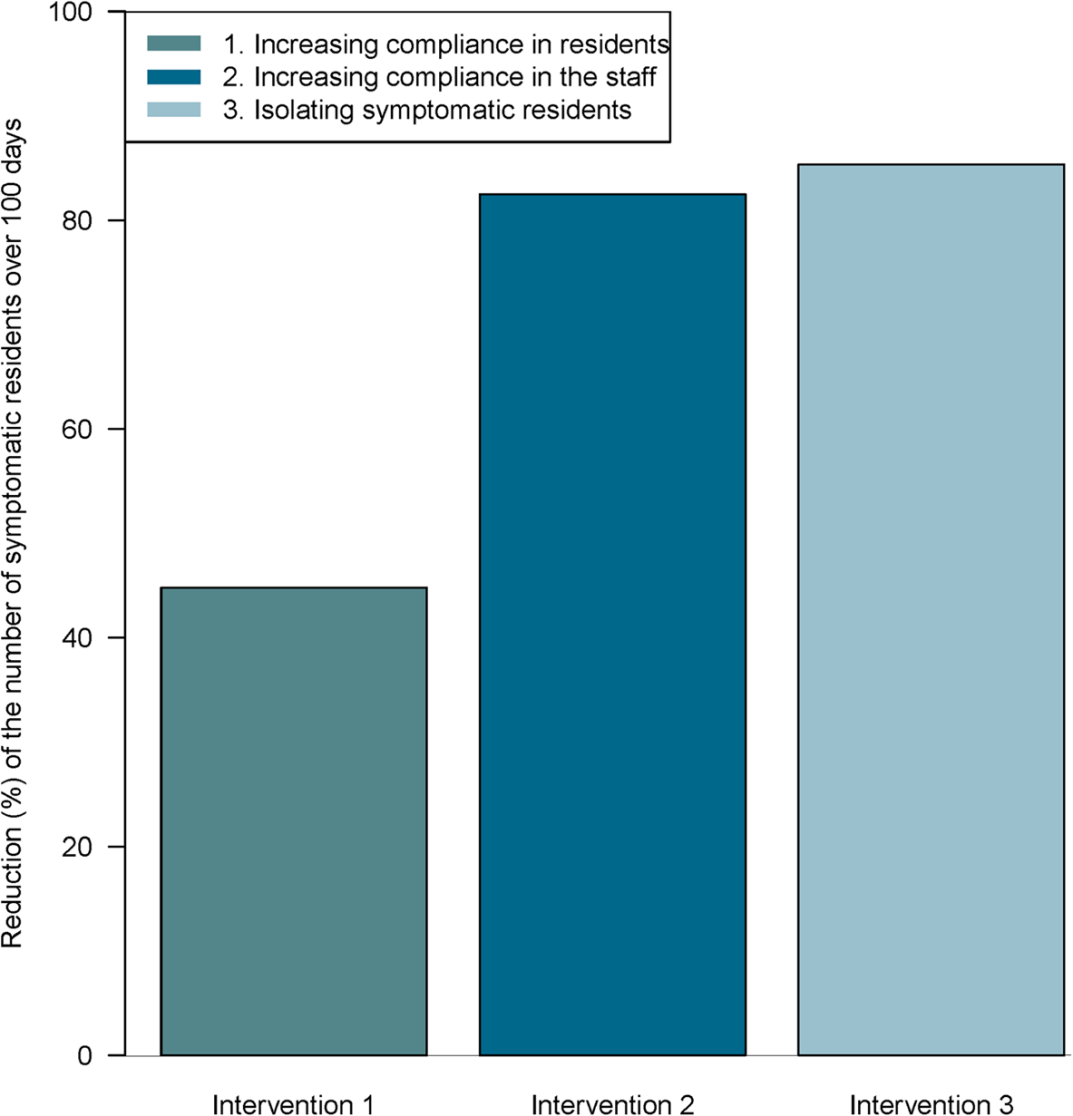
Comparison of three control strategies: predicted reduction in the cumulated number of norovirus infection cases among residents over 100 days due to three interventions: resident HH compliance increased to 60 %, staff HH compliance during staff-resident contacts increased to 60 %, and isolation of infected residents

Based on our analysis of the impact of hand hygiene, three control strategies were compared with the baseline scenario to assess their effectiveness in controlling norovirus spread in NHs (Fig. 5): increasing resident hand hygiene compliance up to 60 %; increasing staff hand hygiene compliance during their contacts with residents up to 60 %; and isolating infected residents from all non-strictly necessary contacts.

Figure 5 depicts the predicted reduction in the number of symptomatic over 100 days when these strategies are implemented, as compared to baseline. The predicted 19 infected residents over 100 days in the baseline scenario decreased to 11 when resident hand hygiene was increased (a 44 % reduction), and to four for control strategies based on either staff hand hygiene or infected resident isolation (an 80 % reduction).

### Sensitivity analyses

**Fig. 6:**
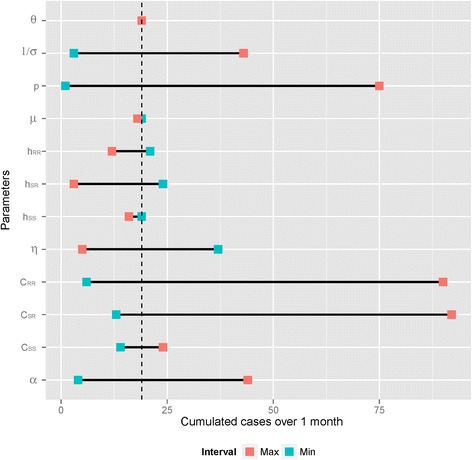
Tornado diagram of the effects of model parameters on the predicted cumulated number of norovirus infection cases among residents over 100 days. Blue squares indicate predictions associated with the lower parameter values; red squares indicate predictions associated with the higher parameter values

The results of the sensitivity analyses are provided in Fig. 6 (tornado diagram - univariate analysis) and Table 3 (PRCC - multivariate analysis). The most important changes in the predicted number of cases were observed with changes in parameters pertaining to the transmission process: the per-contact transmission probability (p), the contact rates between residents (C_RR_) and between residents and staff (C_SR_), and the relative infectiousness during phases A and E (α) were all significantly positively correlated with epidemic size. Additionally, the number of cases increased with the average duration of symptoms (1/σ) and decreased when the proportion of diagnosed infected staff (η) increased. Finally, the impact of staff hand hygiene compliance during their contacts with residents (h_SR_) was confirmed.

**Table 3:** Results from the multivariate sensitivity analysis: key factors that increase (Partial Rank Correlation Coefficient [PRCC] > 0) or decrease (PRCC < 0) the predicted total number of resident cases over 100 days. A higher absolute value of PRCC indicates a strong relationship between that parameter and the outcome

Parameters			PRCC
Admission and discharge rate		μ	0.085176
Transmission probability		p	0.881686
Detection rate of infected staff		η	−0.444676
Contact rate	Staff-Staff	C_SS_	0.143079
	Staff-Resident	C_SR_	0.428949
	Resident-Resident	C_RR_	0.902967
Relative infectiousness of A and E		α	0.697669
Duration of symptoms		1/σ	0.767952
Rate of immunity loss		θ	0.050308
Hand hygiene compliance rate during interactions	Staff-Staff	h_SS_	−0.164771
	Staff-Resident	h_SR_	−0.539334
	Resident-Resident	h_RR_	−0.563288

## Discussion

In this work, we developed a mathematical model to analyze norovirus spread in NHs, to study the impact of hand hygiene on this spread and to compare the effectiveness of various control strategies. Our results have potential practical implications for long-term care staff and managers. First, they confirm the major importance of hand hygiene practices among staff members in contact with residents for the prevention of gastroenteritis epidemics. In addition, they provide insight regarding the optimal hand hygiene compliance rate that should be aimed for. Indeed, based on our predictions, increasing up to 60 % the hand hygiene compliance among NH staff during their contacts with residents may help control norovirus outbreaks very effectively, but further increases in this compliance will not bring a major improvement in norovirus control. Knowing that the average observed hand hygiene compliance rate in NHs is only about 15 % [6], this suggests that NH staff members should be more compliant to international recommendations. Our results also suggest that isolating infected residents from other residents and from unnecessary staff contacts is a highly effective control strategy.

Nevertheless, the implementation of infection control measures in NH settings may be hindered by several factors. First, some measures are too costly for NHs. This could include long-term hand hygiene promotion interventions. Second, staff education is made more difficult by a high turnover. This explains in part the low level of observed hand hygiene compliance, and may lead to difficulties in increasing this compliance to 60 %. Third, NHs are, as well as healthcare facilities, home to their residents. Hence, a reluctance to implement some control measures in NHs is sometimes reported, as staff members feel that they are not adapted to long-term care and tend to over-medicalize resident-staff contacts. This may apply to the two strategies we identified as most efficient: increased hand hygiene during staff-resident contacts and infected resident isolation.

Additionally, this study has several limitations, which are discussed next.

First and foremost, while norovirus is known to have a high persistence in the environment, the contamination of the NH environment by norovirus was not explicitly taken into account in the model, due to the lack of available data. Rather, the risk of norovirus acquisition from the environment was indirectly modeled within the between-individual contact rates C_RR_, C_SR_ and C_SS_. This may have led us to over-estimate the role played by inter-individual transmission in norovirus spread dynamics, and, in turn, to over-estimate the impact of interventions which aim at reducing the transmission risk, such as hand hygiene and resident isolation. Indeed, only 25 % of published randomized trials found a significant impact of hand hygiene on the infectious risk in nursing homes [7]. Conversely, other interventions, such as hand hygiene performed by the staff after contacts with the residents’ environment, could probably have been found to have an important impact on norovirus dynamics.

Second, not including possibly infected visitors to the NH, as well as the admission of infected residents, led to other neglected pathways for norovirus acquisition. However, even at the epidemic peak, the observed prevalence of gastroenteritis in French adults is approximately 3 %, which would have translated in a very low risk of introduction to a given NH via these pathways.

Third, observed data suggests that up to 30 % of healthy adults infected by norovirus may be asymptomatic. While the SEIAR model has been shown before to reproduce norovirus epidemics in a satisfactory manner [8], it does not explicitly allow for totally asymptomatic infections. Hence, we included the possibility for infected staff members to present only very light to non-detectable symptoms, via the probability η of infection detection in staff members.

Fourth, sensitivity analyses showed that contact rates involving the staff had a major impact on norovirus spread within the NH. While the contact rates we used were based on observed data collected during a recent survey, they were still aggregated indicators which did not account for the real-life variability of contact patterns among the staff. In future work, developing an individual-based model would allow us to describe in more detail the timetables and contacts of each individual staff member working in the NH. This would allow us to take into account heterogeneities among residents in terms of care needs and susceptibility to norovirus infection.

## Conclusions

This work provides an operational approach to simulate and better understand norovirus spread in NH settings. Based on a recent review, there is a strong need for more studies on the impact of hand hygiene-based interventions in long-term care, in particular randomized trials [7]. Because it allows assessing the impact of a wide range of control strategies in a fast and simple manner, the modeling approach we propose is a useful complement to such much-needed studies.

## Abbreviations

HH, hand hygiene; NH, nursing home; SEIAR, susceptible, exposed, infected, asymptomatic, recovered

## Appendix

**Table 4:** List of the model stochastic transitions and their rates

Events	Transitions	Rates
**1**- Resident admission to the NH	S_*R*_ → S_*R*_ + 1	*μ* × N_*R*_
**2**- Susceptible resident discharge out of the NH (or death)	S_*R*_ → S_*R*_ − 1	*μ* × S_*R*_
**3**- Susceptible resident exposed to the virus	S_*R*_ → S_*R*_ − 1 & E_*R*_ → E_*R*_ + 1	*⋋*_*R*_ = p × [(1-h_*RR*_) × C_*RR*_×(I_*R*_ + α × E_*R*_ + α × A_*R*_) + (1-h_*SR*_) × C_*SR*_×(I_*S*_ + α × E_*S*_ + α × A_*S*_)]
**4**- Exposed resident discharge out of the NH (or death)	E_*R*_ → E_*R*_-1	*μ* × E_*R*_
**5**- Start of symptoms in exposed residents	E_*R*_ → E_*R*_-1 & I_*R*_ → I_*R*_ + 1	_*∈*_ × E_*R*_
**6**- Infected symptomatic resident discharge out of the NH (or death)	I_*R*_ → I_*R*_-1	*μ* × I_*R*_
**7**- Asymptomatic resident discharge out of the NH (or death)	A_*R*_ → A_*R*_-1	*μ* × A_*R*_
**8**- End of symptoms in residents	I_*R*_ → I_*R*_-1 & A_*R*_ → A_*R*_ + 1	*σ* × I_*R*_
**9**- Recovery of an asymptomatic resident	A_*R*_ → A_*R*_-1 & R_*R*_ → R_*R*_ + 1	*ρ* × A_*R*_
**10**- Recovered resident discharge out of the NH (or death)	R_*R*_ → R_*R*_-1	*μ* × R_*R*_
**11**- Immunity loss of a resident	R_*R*_ → R_*R*_-1 & S_*R*_ → S_*R*_ + 1	*θ* × R_*R*_
**12**- Susceptible staff member exposed to the virus	S_*S*_ → S_*S*_-1 & E_*S*_ → E_*S*_ + 1	*⋋*_*S*_ = p × [(1-h_*SS*_) × C_*SS*_×(I_*S*_ + α × E_*S*_ + α × A_*S*_) + (1-h_*SR*_) × C_*SR*_×(I_*R*_ + α × E_*R*_ + α × A_*R*_)]
**13**- Start of symptoms in exposed staff members	E*p* → E_*S*_-1 & I*p* → I_*S*_ + 1	_*∈*_ × E_*S*_
**14**- End of symptoms in staff members	I_*S*_ → I_*S*_-1 & A_*S*_ → A_*S*_ + 1	*σ* × I_*S*_
**15**- Recovery of an asymptomatic staff member	A_*S*_ → A_*S*_-1 & R_*S*_ → R_*S*_ + 1	*ρ* × A_*S*_
**16**- Immunity loss of a staff member	R_*S*_ → R_*S*_-1 & S_*S*_ → S_*S*_ + 1	*θ* × R_*S*_

S_*R*_ and S_*S*_ respectively number of susceptible residents and staff members

E_*R*_ and E_*S*_ respectively number of exposed residents and staff members

I_*R*_ and I_*S*_ respectively number of infected symptomatic residents and staff members

A_*R*_ and A_*S*_ respectively number of asymptomatic residents and staff members

R_*R*_ and R_*S*_ respectively number of recovered residents and staff members

*μ* admission and discharge rate

*p* per-contact probability of transmission

C_*RR*_ contact rate resident-resident

C_*SR*_ contact rate staff-resident

C_*SS*_ contact rate staff-staff

h_*RR*_ hand hygiene compliance rate during resident-resident interactions

h_*SR*_ hand hygiene compliance rate during staff-resident interactions

h_*SS*_ hand hygiene compliance rate during staff-staff interactions

α Relative infectiousness of A and E individuals

1/_*∈*_ Duration of incubation

1/*σ* Duration of symptoms

*ρ* Recovery rate

*θ* Rate of immunity loss
